# Epidemiological Characteristics of COVID-19 Outbreak in Yangzhou, China, 2021

**DOI:** 10.3389/fmicb.2022.865963

**Published:** 2022-05-06

**Authors:** Yu-Dong Zhang, Ding Chen, Lei Hu, Liang Shen, Ren-Yuan Wu, Fu-Ming Cao, Jian-Qiang Xu, Liang Wang

**Affiliations:** ^1^The First Clinical Medical College of Xuzhou Medical University, Xuzhou, China; ^2^School of Medical Informatics and Engineering, Xuzhou, China; ^3^School of Mathematics, China University of Mining and Technology, Xuzhou, China; ^4^School of Management, Xuzhou Medical University, Xuzhou, China; ^5^School of Medical Imaging, Xuzhou Medical University, Xuzhou, China; ^6^The Second Clinical Medical College of Xuzhou Medical University, Xuzhou, China

**Keywords:** COVID-19, SARS-CoV-2, epidemiology, outbreak, basic reproduction number

## Abstract

**Objective:**

Epidemiological characteristics of COVID-19 outbreak in Yangzhou city caused by the highly contagious Delta variant strain of SARS-CoV-2 virus were investigated in this retrospective descriptive study to provide prevention and control guidelines for outbreaks in the future.

**Methods:**

All the epidemiological data used in this study were collected manually from the official website of the Yangzhou Municipal Health Committee from 28 July to 26 August 2021, and then were analyzed systematically and statistically in this study.

**Results:**

A total of 570 COVID-19 cases were reported during the short-term outbreak in Yangzhou City. The ages of infected individuals ranged from 1 to 90 years with the average age at 49.47 ± 22.69 years. As for gender distributions, the ratio of male- to-female patients was 1:1.36 (242:328). Geographic analysis showed that 377 patients (66.1%) were in Hanjiang District while 188 patients (33.0%) were in Guangling District. Clinical diagnosis showed that 175 people (30.7%) had mild symptoms, 385 people were in moderate conditions (67.5%), and 10 people were in severe situations (1.8%). Significant age differences were found among the three groups (*P* < 0.001). However, no significant difference was identified in terms of gender ratio (*P* > 0.05). Based on the transmission chain formed by 6 generations of infected persons with a clear transmission relationship, the age showed a gradually decreasing trend, while the median time of diagnosis in 2 adjacent generations was 3 days. In addition, the estimated basic reproduction number *R*_0_ of the Delta variant was 3.3651 by the classical Susceptible, Infectious, and/or Recovered (SIR) model.

**Conclusion:**

The Delta variant of SARS-CoV-2 was highly infectious and has obvious clustering characteristics during the Yangzhou outbreak in China.

## Introduction

Coronavirus disease 2019 (COVID-19) caused by the severe acute respiratory syndrome coronavirus 2 (SARS-CoV-2) has been widely spread worldwide since the end of 2019 (Poon and Peiris, 2020), resulting in a serious impact on human health and global economy. As of now, according to the Coronavirus Resource Center at Johns Hopkins University & Medicine,^1^ the number of confirmed COVID-19 patients worldwide has exceeded 488 million and the death toll has exceeded 6.14 million, and the administration of vaccine doses have exceeded 10.88 billion (World Health Organization, 2020). With the accelerating mutation rates of the SARS-CoV-2 virus, new variant strains, such as α, β, γ, δ, and ε, are constantly emerging (Boehm et al., 2021; Giovanetti et al., 2021), not even mentioning the recently emerged highly contagious Omicron strain. The spread and pathogenicity of these mutant strains in the population showed an increasing trend (Janik et al., 2021; Plante et al., 2021; Yi and Wenhong, 2021), leading to the continued spread of COVID-19 worldwide. Therefore, the current international epidemic situation is still complex and severe. As for the worldwide model of COVID-19 prevention and control due to the low infection and death rates, the experience from China has high values of practical guidance. With the efforts of medical staff to actively participate in epidemic control, the epidemic situation of COVID-19 has been effectively controlled. However, due to the uncertainties of SARS-CoV-2 transmission, epidemic still occurs sporadically in local areas. In the face of the increasingly severe epidemic situation abroad and the great import pressure of confirmed cases from foreign countries, there have been short-term and small-scale COVID-19 outbreaks in China recently. Since 28 July 2021, the outbreak of COVID-19 in Yangzhou has been caused by related cases in the Nanjing Lukou International Airport. After nearly a month of hard work, the epidemic was effectively controlled. According to the epidemiological data reported by the Yangzhou Health Commission,^2^ the situation of the epidemic situation was preliminarily analyzed and reported in this study.

## Materials and Methods

### Data Collection

All the data Son COVID-19 in Yangzhou came from the epidemic report issued on the public official website of the Yangzhou Commission of Health and were released by the Yangzhou Municipal Government’s Press from 28 July to 26 August 2021, all of which were deidentified and available publicly. Information, such as personnel number, age, sex, address, diagnosis, date of diagnosis, and close contacts of each confirmed case, was collected from the public website. Therefore, no informed consent forms and ethics approval were needed for this study.

### Analysis of Epidemic Characteristics

All the COVID-19 cases were collected and constructed into a formatted dataset. A descriptive epidemiological method was adopted to analyze the time, place, and population characteristics of COVID-19 in Yangzhou from 28 July to 26 August 2021.

### Preliminary Discussion on Factors Affecting Disease Classification in COVID-19

There were three types of infections for the disease, that is, mild, common, and severe, based on the Diagnosis and Treatment Protocol for the COVID-19 patients jointly released by the National Health Commission of the People’s Republic of China and the National Administration of Traditional Chinese Medicine (Trial Version Eight; National Health Commission, 2022). In this study, all the patients were divided into three groups according to disease types that was confirmed by the Yangzhou Municipal Government *via* their daily-released public report, which were then compared through differences in genders, ages, and addresses.

### Analysis of the Spread of COVID-19 in the Population

We sorted out the transmission chains of all confirmed cases during Yangzhou Outbreak according to the information in the epidemic notification updated daily by the Yangzhou Municipal Government. The confirmed cases from Jiangning District in Nanjing (the Capital City of Jiangsu Province that was adjacent to Yangzhou) were designated as the 0th generation of infected persons, while the confirmed cases in close contact with the 0th generation of infected persons were recorded as the 1st generation of infected persons, and so on and so forth. The number and basic information of infected persons in each generation were enumerated and recorded, leading to the formation of a complete and clear transmission chain.

### Mathematical Model of the Spread of COVID-19

The classic Susceptible, Infectious, and/or Removed (SIR) epidemic model for infectious diseases proposed by Kermack and McKendrick (1927) was used to mathematically model the current round of COVID-19 transmission chain in Yangzhou in this study. In the SIR model, S, I, and R correspond to the numbers of susceptible, infected, and removed people, respectively. The rules for the dynamic changes in the numbers of the three groups of people over time could be expressed by the following ordinary differential Equations 1–3:


$$\frac{d\,S}{d\,t}=-\beta \,\frac{S\,I}{N}\;\left(1\right)$$

$$\frac{d\,I}{d\,t}=\beta \,\frac{S\,I}{N}-\gamma \,I\;\left(2\right)$$

$$\frac{d\,R}{d\,t}=\gamma \,I\;\left(3\right)$$

where *N* indicates the total number of people, and β is the infection rate of susceptible people in contact with infected persons, and γ is the average recovery rate of the infected population, which is a fixed value that depends on the average duration of infection.

Then, we made determinations of the main parameters in the equations. The initial values of S, I, and R were set to 1, *N* - 1, and 0, respectively, where *N* was the total population of Yangzhou City. γ was the recovery rate. The recovery period of the COVID-19 epidemic was about 30 days, so γ was set to be 1/30. To facilitate the identifications of parameters, we then made a simplification of the above SIR model. Since the number of patients in the early stage of transmission was small, it was approximated that all people were susceptible people, that is, S≈N. Thus, Equation 2 was re-formulated as Equation 4 here:


$$\frac{d\,I}{d\,t}=\left(\beta -\gamma \right)\,I\;\left(4\right)$$

Through Equation 4, it was convenient to know that the general solution of the differential equation was:


$$I\,\left(t\right)=C\,e^{\left(\beta -\gamma \right)\,t}\;\left(5\right)$$

where C was a constant. C was equal to 1 since *I*(*t* = 0) = 1. Thus, Equation 5 could be written as:


$$I\,\left(t\right)=e^{\left(\beta -\gamma \right)\,t}\;\left(6\right)$$

Therefore, the following parameter identification problem could be constructed:

Decision variable: infection rate β

Objective function: $m\,i\,n\,\sum_{t\in T}{\left(e^{\left(\beta -\gamma \right)\,t}-\hat{I}\,\left(t\right)\right)}^{2}\;\left(7\right)$

where $\hat{I}$ was the actual number of patients (real-world data) and *t* was the time set in a day. Our model could be hypothesized as follows: a sick person was in close contact with 5 people every day and the recovery rate γ = 1/30 and N≈S in the early stage of the epidemic. The infection rate of COVID-19 could be obtained by solving the aforementioned optimization problem.

The infection rate could be written as β = nContact × infecProb, where nContact was the number of uninfected people that infected people came into contact with each day, and infecProb was the infection probability. The basic reproduction number of COVID-19 could be estimated based on the following Equation 8:


$$R_{0}=\frac{\beta }{\gamma }\;\left(8\right)$$

### Statistical Analysis

The software SPSS 20.0 was used for the statistical analysis. *$\bar{x}$* ± *s* was applied to describe the measurement data, where $\bar{x}$ indicated the mean and *s* indicated standard variance. Frequency and composition were used to compare the counting data. In the analysis of influencing factors of disease typing, the non-parametric test was applied if the measurement data did not meet the homogeneity of variance, and count data were tested using the χ^2^ test or Fisher’s test.

## Results

### General Information

**FIGURE 1:**
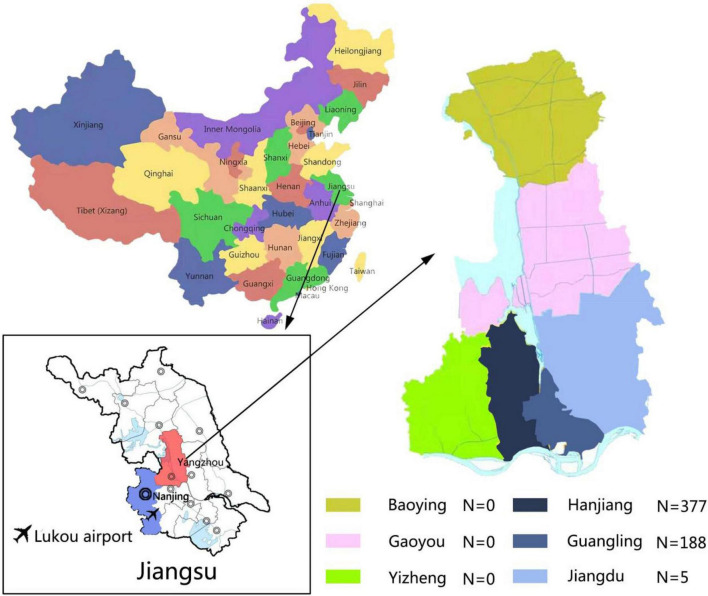
Schematic illustration of the Yangzhou City geography and the corresponding distribution of confirmed cases in different districts of Yangzhou City. A total of six districts in Yangzhou City were present with confirmed COVID-19 cases, which were Baoying (*N* = 0), Gaoyou (*N* = 0), Yizheng (*N* = 0), Hanjiang (*N* = 377), Guangling (*N* = 188), and Jiangdu (*N* = 5).

Yangzhou is located in the middle of Jiangsu Province, and its southwest border is adjacent to Nanjing as shown in Figure 1. From 28 July to 26 August 2021, Yangzhou had reported 570 confirmed cases, including 242 men (42.5%) and 328 women (57.5%) with an average age of 49.47 ± 22.69 years. After grouping by age, there were 77 (13.51%) within 0–18 years old, 122 (21.40%) within 19–40 years old, 145 (25.44%) in 41–60 years old, and 226 (39.65%) over 61 years old, of which the elderly over 61 years old accounted for the most. The geographic distribution of confirmed cases in Yangzhou based on their addresses was as following: 188 people (33.0%) in Guangling district, 377 people (66.1%) in Hanjiang District, and 5 people (0.9%) in Jiangdu district, of which the number of confirmed cases in Hanjiang District accounts for more than half of the total confirmed cases. According to the COVID-19 guideline (National Health Commission, 2022), the confirmed cases consisted of 175 (30.7%) in mild type, 385 (67.5%) in common type, and 10 (1.8%) in severe type. Thus, the common type had the largest ratio of confirmed cases.

**FIGURE 2:**
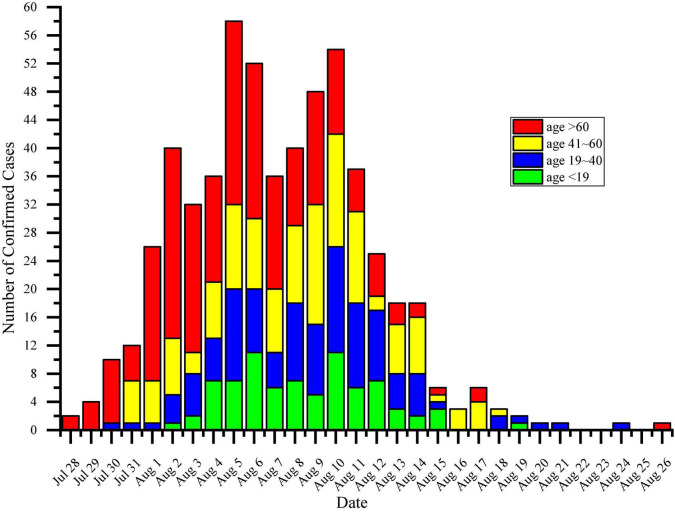
Quantitative visualization of the daily diagnostic number of COVID-19 in Yangzhou, China. The compositions of confirmed cases in different age groups were characterized by cumulative bars in different colors. Green bar: < 19 years old. Blue bar: between 19 and 40 years old. Yellow bar: between 41 and 60 years old. Red bar: > 60 years old.

The time distribution of the number of confirmed patients per day was further investigated, which was sorted according to the date of diagnosis in the daily report by the Yangzhou Municipal Government. In particular, the time of diagnosis was mainly from 28 July to 26 August, with the peak period from 1 to 14 August. The number of confirmed patients showed an obvious upward trend, peaked, and then fluctuated down. The largest daily confirmed number appeared in 5 August. In terms of age composition for the confirmed cases per day, the proportion of people over 61 years old group was significantly higher than that in other age groups in the early stage of the epidemic (before 8 August 2021), then gradually decreased until the epidemic was over. The changing pattern of the COVID-19 patients was visualized in details in Figure 2.

### Preliminary Study on Influential Factors of COVID-19 Disease Classification

The differences in age distribution, gender distribution, and regional distribution in the three groups of the mild type, common type, and severe type of confirmed the COVID-19 cases were analyzed by statistical methods. The results were shown in Table 1. Since the age data of the three groups did not meet the homogeneity of variance, the non-parametric test was used for the analysis of the age difference. The average age rank of the mild group was smaller, while the average age rank of the severe group was larger. The results showed that the age of the mild group was lower while the age of the severe group was higher (*H* = 3.177,*P* < 0.001). There was no significant difference in gender composition among the three groups by the chi-squared test (χ^2^ = 3.177,*P* = 0.204). The statistical results showed that the gender composition of patients with mild, common, and severe COVID-19 was similar in the three groups. In terms of regional distribution, the number of patients in Jiangdu district was significantly less than that in Hanjiang District and Guangling district. Statistical analysis by the Fisher’s exact probability method showed that the composition of the three groups was different (*P* value = 0.029). Therefore, of all the patients in Yangzhou, common and severe cases were mainly distributed in the Hanjiang District.

**TABLE 1:** Differences in age, gender, and regional distribution of the confirmed COVID-19 patients with different disease types.

Classification	Age			Gender		Region		
	(*$\bar{x}$* ± *s*)	Cases	Rank	Male	Female	Hanjiang	Guangling	Jiangdu
Mild type	35.29 ± 23.26	175	188.30	84	91	102	70	3
Common type	55.17 ± 1 9.30	385	323.69	154	231	266	117	2
Severe type	77.90 ± 6.30	10	516.10	4	6	9	1	0
Statistic			101.30		3.177		10.516	
*P*-value			<0.001		0.204		0.029	
								

### Analysis of the Spread of COVID-19 in Population

According to the reported contact relationship in the epidemic notification, a complete and clear COVID-19 transmission chain was sorted out, with a total of 198 infected patients in 6 generations. The number of confirmed patients, average age, gender composition, address distribution, disease classification, and median date of diagnosis of each generation were shown in Table 2. Among the 6 generations of confirmed patients with clear infectious relationships, the age tended to decrease gradually. The median diagnosis time between the adjacent 2 generations was around 1–5 days, and the median time was 3 days.

**TABLE 2:** Basic information of communication chain formed by six generations of confirmed patients.

Generation	Cases	Age (years)	Gender (Male/Female)	Address (Hanjiang/Guangling/Jiangdu)	Classification (Mild/Common/Severe)	Median Date of Diagnosis
0th	1	64	0/1	1/0/0	0/1/0	7/28
1st	66	70.17 ± 9.22	17/49	64/2/0	11/50/5	8/2
2nd	68	55.0 ± 21.32	30/38	54/13/1	23/43/2	8/4
3rd	41	46.65 ± 24.93	17/24	19/22/0	20/20/1	8/5
4th	15	29.47 ± 20.03	8/7	9/6/0	9/6/0	8/8
5th	6	23.0 ± 16.27	3/3	4/2/0	2/4/0	8/11
6th	1	40	0/1	0/1/0	0/1/0	8/13

### The Mathematical Model of COVID-19 Communication

**FIGURE 3:**
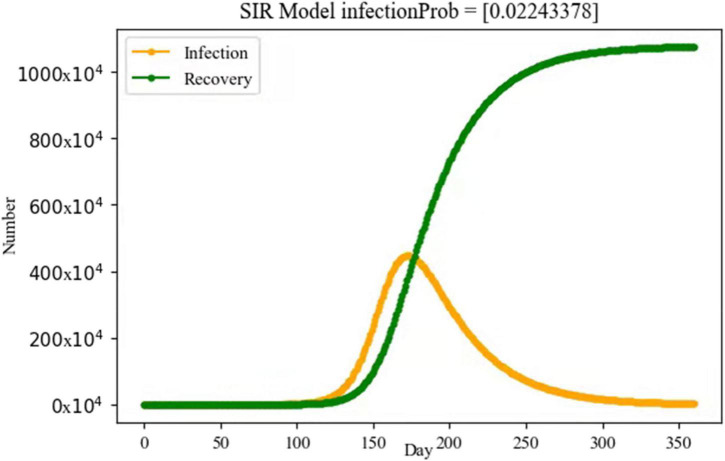
Simulation of the numerical change curve through SIR model for the Yangzhou epidemic. Yellow line: number of infected persons. Green line: number of recovery persons.

Through SIR model, we calculated the identification result as infecProb = 0.02243 and infection rate as β = 0.11217. The basic reproduction number of COVID-19 was estimated to be *R*_0_ = 3.3651. The results showed that a person infected with COVID-19 would transmit the disease to 3.37 susceptible persons on average during the Yangzhou epidemic. Figure 3 showed the numerical change curve of SIR model for the Yangzhou epidemic.

## Discussion

The first confirmed COVID-19 case was reported in Yangzhou on 28 July 2021 and the epidemic was effectively controlled on 26 August 2021. Thus, the newly emerging COVID-19 outbreak in Yangzhou lasted for less than a month before completely and timely control of the situation. Center for Disease Control and Prevention of Jiangsu province confirmed that the virus strain circulating in Yangzhou was Delta B.1.617.2 variant (Yangzhou Municipal People’s Government, 2021; Hu et al., 2022). SARS-CoV-2 of Delta type initially emerged in India in October 2020 (Cascella et al., 2022) and was one of the five variants of concerns (VOCs) by the WHO. So far, the Delta strain has caused epidemics in many countries and regions around the world. Mutations in the Delta-type virus strain can lead to an increased affinity between the spinous process protein and the ACE 2 receptor, thereby enhancing viral adhesion and subsequent entry into host cells (Cascella et al., 2022). COVID-19 caused by this strain is characterized by high viral load, rapid replication *in vivo*, rapid transmission, strong infectivity, and high virulence, which leads to shortened incubation period of the disease (Du et al., 2021; Hu et al., 2022), certain immune escape ability of the virus (Mlcochova et al., 2021), and higher hospitalization rate of the infected (Callaway, 2021). As a result, the main urban area of Yangzhou broke out with COVID-19 in a short period of time.

Nevertheless, the clinical symptoms of patients infected with the Delta variant strain appeared to be less severe than those of the wild-type strain. Hu et al. compared the inpatients during the epidemic in Yangzhou with those during the epidemic in Wuhan in 2020, and found no difference in gender, age, and the prevalence of underlying diseases, such as hypertension, diabetes, and cardiovascular disease, between the two groups (Hu et al., 2022). The study found that patients infected with the Delta variant SARS-CoV-2 had a significantly lower frequency of major clinical symptoms, such as cough and fever than those infected with the wild-type strain. However, a new symptom of sore throat was found in patients infected with the Delta variant (Hu et al., 2022). Epidemiological survey information showed that among the confirmed cases of the epidemic in Yangzhou, there were more elderly people older than 61 years, and the 10 severe patients were all over 70 years. In the early stage of the epidemic, the characteristics of site aggregation infection were obvious. There were a large number of elderly patients who were infected and had not formed effective immune protection, resulting in some severe cases among infected people. From April to July, the Center for Disease Control and Prevention (CDC) conducted an epidemiological survey of more than 600,000 COVID-19 cases in the United States mainland, the result of which showed that 569,142 cases (92%) had not completed all vaccination procedures and 46,312 cases (8%) had completed all vaccination procedures; among those hospitalized with COVID-1,9 34,972 (92%) unvaccinated and 2,976 (8%) vaccinated were completed; among people who died from COVID-19, 6,132 (91%) were unvaccinated and 616 (9%) were vaccinated (Scobie et al., 2021). As a result, the risk of contracting COVID-19 was about 10 times higher for people who had not completed the full vaccination process. Therefore, vaccination was an effective means of preventing COVID-19 infection and reducing hospitalizations and mortality.

Previous studies had shown that the median incubation period of COVID-19 was about 5.1 days (95% CI, 4.5–5.8 days; Lauer et al., 2020), and transmission could occur during the incubation period (Liu et al., 2020; Yu et al., 2020). Most patients developed symptoms within 11.5 days after infection (Lauer et al., 2020). The difference was that the mean incubation period of the Delta strain of COVID-19 was only 4.4 days (Zhang et al., 2021), and the mean interval between generations was 2.9 days. By comparing the median diagnosis time of 6 generations of infected persons in Yangzhou, it was found that the median diagnosis time of 2 adjacent generations was only 3 days. The passage interval of the disease was significantly shortened and the virus was spread much faster. In the terms of human transmission, the epidemic was mainly among the elderly at the beginning and gradually spread to the general population. As can be seen from Table 1, in the early stage of the epidemic, the daily number of confirmed cases and the relative proportion of elderly people aged over 61 were significantly higher than those of other age groups, and gradually decreased after 8 August. For other age groups, the daily number of confirmed cases increased significantly after 4 August, and the overall number also showed a trend of first increment and then decrement. In addition, there was an overall decrease in the age of each generation in the transmission chain consisting of six generations of the confirmed patients with clear contacts.

The basic reproduction number is an important indicator to measure the infectiousness of an infectious disease. Using the SIR transmission model of infectious diseases, combined with the data reported in the Yangzhou outbreak, the *R*_0_ of Delta variant of SARS-CoV-2 in this outbreak was calculated as 3.3651. The results indicated that one person infected with COVID-19 in the Yangzhou outbreak would transmit the disease to an average of 3.37 susceptible persons. Wang et al. (2020) evaluated the basic reproductive number of COVID-19 in China and obtained *R*_0_ was equal to 3.49 with 95% CI at 3.42–3.58. Yu et al. (2020) analyzed cluster outbreaks caused by Delta mutant strains in Guangdong Province and calculated that *R*_0_ was about 3.2, significantly higher than that of wild strains. Liu et al. (2021), based on model studies, found that the *R*_0_ of the Delta variant strain was about 6 in the absence of ideal transmission of any intervention. A study from the United Kingdom on the transmissibility of the Delta mutant strain estimated *R*_0_ to be about 7 (Burki, 2021). These results suggest that the transmissibility of the Delta variant is significantly higher than that of the Alpha variant.

Until now, China has experienced several local, short, small-scale COVID-19 outbreaks. However, with the intervention of the government’s effective epidemic prevention policy, the big data method has been used to quickly lock and isolate the infected and close contacts. The government also carried out large-scale nucleic acid screenings as soon and as often as possible (Li et al., 2021), strengthened the control of epidemic-affected communities, and enhanced public health measures at the community level (Li and Gao, 2020). Early detection, early reporting, early isolation, and early treatment of infected persons also contributed to the quickly control of and shorten the duration of the epidemic. As a result, the epidemic in all regions of China has been brought under control quickly and efficiently, which made the infected cases rarely spread to other areas. However, there were also some limitations to the study. At first, the data were all from the epidemic notification issued by the Health Commission of Yangzhou and the Yangzhou Municipal Government, which could only represent the infected population in Yangzhou. Second, the amount of collected information was relatively small, so the research on diseases was not comprehensive and detailed enough. Taking disease typing as an example, the ten severe cases sorted out in this study were diagnosed by the doctors initially without considering the development and outcome of later diseases. Thus, a part of the confirmed patients who turned into severe cases in the treatment process was not included, which might be inconsistent with the total number of severe patients sorted out after the epidemic. Therefore, to increase the accuracy and reliability of research results, subsequent studies were definitely needed to include data from multiple regions for an integrative analysis.

## Conclusion

In this study, we thoroughly sorted out the transmission chains of all confirmed cases in Yangzhou, which was currently the only complete epidemiological analysis of the COVID-19 outbreak in Yangzhou. The Delta variant of SARS-CoV-2 in the Yangzhou outbreak was also highly contagious as expected. There were a large number of COVID-19 infected patients in the age group greater than 61 years old, and the main type of the disease was classified as common with a certain number of severe cases. The interval time between the diagnosis of two generations of patients was significantly shortened, and the spreading rate of the virus was increased. Therefore, the experience of the epidemic situation in Yangzhou was that, by executing scientific and accurate prevention and control strategies, carrying out large-scale nucleic acid screening and community control as soon as possible, and by isolating and treating infected persons, can the epidemic situation be quickly contained with less casualties at a municipal level, which could also provide references for better control and prevention of the COVID-19 outbreak in other regions.

## Data Availability Statement

The original contributions presented in the study are included in the article/supplementary material, further inquiries can be directed to the corresponding author.

## Ethics Statement

All the data were deidentified and publicly accessible. There is no need to require ethical approval for the study.

## Author Contributions

Y-DZ, LW, and J-QX contributed to the design of the research. F-MC and Y-DZ collected and sorted out the raw data. R-YW and J-QX carried out the statistical analysis of the data. LH and LS constructed the SIR model. Y-DZ and DC completed the first draft of the article. All authors critically revised and approved the submitted version.

## Conflict of Interest

The authors declare that the research was conducted in the absence of any commercial or financial relationships that could be construed as a potential conflict of interest.

## Publisher’s Note

All claims expressed in this article are solely those of the authors and do not necessarily represent those of their affiliated organizations, or those of the publisher, the editors and the reviewers. Any product that may be evaluated in this article, or claim that may be made by its manufacturer, is not guaranteed or endorsed by the publisher.

## Footnotes

1
https://coronavirus.jhu.edu/map.html

2
http://wjw.yangzhou.gov.cn/yzwshjh/ywkd/wjw_list.shtml
